# Diversity of T Cell Epitopes in *Plasmodium falciparum* Circumsporozoite Protein Likely Due to Protein-Protein Interactions

**DOI:** 10.1371/journal.pone.0062427

**Published:** 2013-05-07

**Authors:** Nagesh R. Aragam, Kelly M. Thayer, Nabi Nge, Irving Hoffman, Francis Martinson, Debbie Kamwendo, Feng-Chang Lin, Colin Sutherland, Jeffrey A. Bailey, Jonathan J. Juliano

**Affiliations:** 1 Division of Infectious Diseases, University of North Carolina School of Medicine, Chapel Hill, North Carolina, United States of America; 2 Program in Bioinformatics and Integrative Biology, University of Massachusetts School of Medicine, Worcester, Massachusetts, United States of America; 3 Department of Immunology & Infection, London School of Hygiene & Tropical Medicine, London, United Kingdom; 4 UNC Project-Malawi, Lilongwe, Malawi; 5 Department of Biostatistics, University of North Carolina, Chapel Hill, North Carolina, United States of America; 6 Division of Transfusion Medicine, University of Massachusetts School of Medicine, Worcester, Massachusetts, United States of America; Tulane University, United States of America

## Abstract

Circumsporozoite protein (CS) is a leading vaccine antigen for falciparum malaria, but is highly polymorphic in natural parasite populations. The factors driving this diversity are unclear, but non-random assortment of the T cell epitopes TH2 and TH3 has been observed in a Kenyan parasite population. The recent publication of the crystal structure of the variable C terminal region of the protein allows the assessment of the impact of diversity on protein structure and T cell epitope assortment. Using data from the Gambia (55 isolates) and Malawi (235 isolates), we evaluated the patterns of diversity within and between epitopes in these two distantly-separated populations. Only non-synonymous mutations were observed with the vast majority in both populations at similar frequencies suggesting strong selection on this region. A non-random pattern of T cell epitope assortment was seen in Malawi and in the Gambia, but structural analysis indicates no intramolecular spatial interactions. Using the information from these parasite populations, structural analysis reveals that polymorphic amino acids within TH2 and TH3 colocalize to one side of the protein, surround, but do not involve, the hydrophobic pocket in CS, and predominately involve charge switches. In addition, free energy analysis suggests residues forming and behind the novel pocket within CS are tightly constrained and well conserved in all alleles. In addition, free energy analysis shows polymorphic residues tend to be populated by energetically unfavorable amino acids. In combination, these findings suggest the diversity of T cell epitopes in CS may be primarily an evolutionary response to intermolecular interactions at the surface of the protein potentially counteracting antibody-mediated immune recognition or evolving host receptor diversity.

## Introduction

The development of a successful malaria vaccine has the potential to significantly reduce the estimated one million deaths a year caused by falciparum malaria. A major concern for vaccine development is the extensive genetic diversity of immunogenic *Plasmodium falciparum* antigens. *P. falciparum* circumsporozoite protein (CS) is a leading candidate antigen [1]–[3] and the recent interim analysis of the Phase III RTS,S/AS01E vaccine trial has shown approximately a 55% and 31% reduction in clinical malaria during the first year among children 5–17 months and 6–12 weeks of age, respectively [2], [3]. However, the CS antigen of RTS,S is comprised of a single variant and the impact of the significant natural genetic variation in CS on vaccine efficacy is still unclear [1], [4]–[6].

Cell mediated immunity is thought to be mediated in part by T cell epitopes in the C terminus of the protein, including the epitopes known as TH2 and TH3 [7]–[9]. These two epitopes are highly polymorphic in natural parasite populations [1], [5]. Understanding what drives this diversity could have a profound impact on improving the design of CS-based vaccines. Many theories about the mechanism of diversification in this region have been proposed. Good et al. suggested that they were maintained by natural selection favoring immune evasion (allele-specific immunity) [10]. This hypothesis was supported by the observation that the number of nonsynonymous nucleotide substitutions was higher than synonymous nucleotide substitutions in parasite populations [11], [12]. On the other hand, recent evidence suggests that among CS isolates in the Gambia, there is only limited evidence of balancing selection, implying minimal allele specific immunity in CS [13]. Diversification may also have been driven by other mechanisms. Indirect evidence for selection on CS has been reported during the malaria transmission cycle [14], [15]. This selection has been supported by population studies and is biologically plausible, as CS is required for oocyst development in the mosquito and is centrally involved in gliding motility of the sporozoite [16], [17]. In addition to the diversity within the epitopes, recent analysis identified non-random associations between TH2 and TH3 epitopes in a parasite population in Kenya, consistent with recent mutations in linkage disequilibrium and/or functional constraints on CS limiting the repertoire of permissible amino acids and their combinations [1]. However, this study only evaluated the dominant alleles in the population and may not completely reflect the potential associations between T cell epitopes within the population. Also, recent studies of the population structure of the gene encoding CS (*pfcsp*) suggest that geographically variable levels of diversity and geographic restriction of specific subgroups may have an impact on the efficacy of malaria vaccines in specific geographic regions [18]. Thus, evaluations of the polymorphisms within and associations between T cell epitopes need to be conducted in varying geographic locations to determine whether previous findings in one or two parasite populations are generalizable.

The crystal structure of the C-terminal region of CS, termed the Thrombospondin type-1 repeat super family (TSR) region, containing TH2 and TH3 was recently published, showing unpredicted protein folding due to the presence of a hydrophobic pocket not found in other TSR domains from paralogous molecules from other organisms [19]. This new insight gained from the crystal structure enables us to more extensively investigation of the impact on protein structure of polymorphisms seen in the TH2 and TH3 epitopes in natural parasite populations and to model altered molecular interactions that may occur due to these changes. Using sequences of the TSR domain in parasite populations from Malawi and the Gambia, the patterns of nucleotide diversity within and between the two populations were evaluated, and haplotype associations between TH2 and TH3 polymorphisms elucidated. We characterize the impact of T cell epitope diversity on protein structure by mapping polymorphisms onto the newly derived crystal structure. Based on these findings, we use structural mapping to evaluate the interactions between epitopes and within epitopes, and an exhaustive point mutagenesis approach to identify any intramolecular structural constraints, as well as those residues under diversifying selection, providing new insight into how and why the described patterns of diversity occur.

## Materials and Methods

### Sequence Data

**Figure 1:**
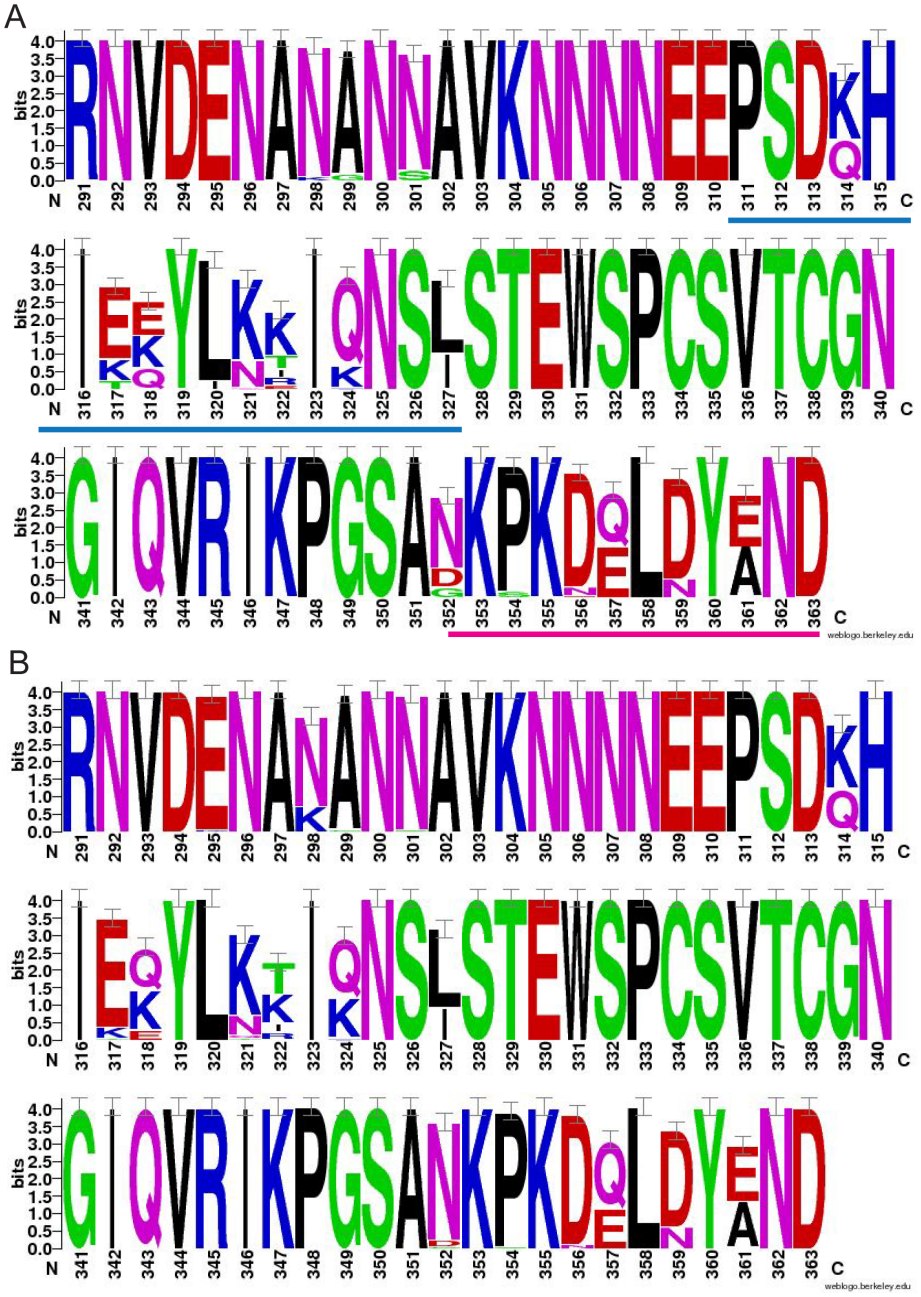
WebLogo of Amino Acid Sequence of Circumsporozoite Protein from Malawi and the Gambia. Panel A and Panel B are the Weblogos for Malawi and the Gambia, respectively. In Panel A, the TH2 region (blue) and TH3 region (pink) are underlined. The TH2 epitope maps almost exclusively to the α-helix, while the TH3 epitope maps to the flap. The polymorphic residues and types of amino acids that populate these sites appear to be conserved between two geographically disparate African parasite populations. Bits represent the information content, which is a relative measurement of sequence conservation, with higher values representing conservation and lower values consistent with sequence diversity at a position.

Sequences from Malawi (GenBank Accession numbers: JN634586– JN634642) were accessed from a previously published study from our group. Details of the sequencing from the 100 participants, which was done by massively parallel pyrosequencing on the 454 platform at University of North Carolina’s High Throughput Sequencing Facility, have previously been published [5]. This deep sequencing allowed for the detection and characterization of minor variants in an infection representing ≥1%. The Gambian *pfcsp* sequences (GenBank Accession numbers: JX885511–JX885521) derive from 55 participants in a clinical trial in the year 2000 [20], [21], and were generated by di-deoxy fluorescent capillary sequencing at The London School of Hygiene & Tropical Medicine (LSHTM). Both major and minor abundance sequence variants from each isolate are reported, where these were unambiguous, as previously described [20]. All sequences from both locales were trimmed to correspond to a 220 bp fragment containing nucleotides 871 to 1090 of PF3d7_0304600 (PlasmoDB, accessed 9/26/2012), corresponding to amino acids 291 to 363 (Figure 1). Consistent with the literature, TH2 was defined as amino acids 311–327 (PSDKHIKEYLNKIQNSL) and TH3 was defined as amino acids 352–363 (NKPKDELDYAND). Written informed consent as approved by The University of North Carolina, Malawian National Health Sciences Research Committee, the Medical Research Council/Gambian Government Joint Ethical Committee, and The London School of Hygiene & Tropical Medicine Ethics Committee was obtained from each participant.

### Data Analysis

DNA alignments for each population were generated by using the DNAStar SeqMan, Version 9.1 [22] and descriptive statistics were generated by DnaSp, Version 5.10.01 [23]. The fixation index (FST) was calculated using Arlequin, Version 3.11 [24]. Neighbor-joining analysis was conducted with MEGA, Version 5 [25]. Bootstrap values, drawn from 500 replicates, were calculated for the deep branch points. Hudson’s nearest-neighbor statistic (Snn) [26] was also calculated for the clustering of samples into geographic clusters. Input was in the form of a pairwise distance matrix between all haplotypes in the phylogenetic tree.

In order to evaluate if there were non-random associations between TH2 and TH3 epitopes within our populations, associations were explored by contingency table. Each unique TH2 and TH3 amino acid sequence was coded and given a unique label (TH2-“x” or TH3-“y”). Paired epitopes were determined by the TH2 and TH3 type that occurred in each sequence haplotype identified in the population. An example is shown in Figure S1. The frequency of each pair of epitopes was then tabulated for each population (Table S1) to determine the observed frequencies of pairings. Due to large number of categories in genotypes, the contingency table was sparse with many zero count cells. To statistically deal with this kind of sparseness, we utilized a log-linear model with Poisson assumption that treats zero counts as sampling zero frequencies [27] implemented within the SAS procedure PROC CATMOD [28]. If non-random associations occurred between TH2 and TH3 types, the distribution of pairings should diverge from the predicted values based solely on the frequencies of the TH2 and TH3 genotypes assuming random association. A significant deviation from non-random pairing of TH2 and TH3 haplotypes was determined based on the overall distribution of the disparity between predicted and observed frequencies using the log-linear model. Additionally, individual tests of each TH2 and TH3 pairing were performed using the log-linear model with Poisson assumption. The significance cutoff was corrected for the number of comparisons by Bonferroni correction.

### Structural Analysis

Structural studies of CS were carried out based on the newly crystallized structure, PDBID 3VDJ [19]. Sequence logos [29] were generated online using WebLogo [30]. The information content (conservation of the sequence) in bits was binned at increments of 0.25 and mapped to the crystal structure via a color scheme indicating the magnitude. Structure figures/images were generated using Visual Molecular Dynamics (VMD) software (University of Illinois at Urbana-Champaign, http://www.ks.uiuc.edu) and rendered with ray tracing in the software PovRay (http://www.povray.org). In order to map a specific haplotype to the structure, the sequencing data provides both the sequence and position, which is then matched to the corresponding position in the structure and evaluated for evidence of interactions between epitopes.

### Point Mutagenesis

Exhaustive independent residue-by-residue point mutagenesis of the CS wild type sequence in the 3VDJ crystal structure was simulated using MUMBO [31] to calculate the Gibbs free energies of the reference and all potential single point mutations from the reference mutant strains. Each amino acid residue in the 3D7 reference structure was mutated to all 19 possible other amino acids one at a time. For each mutation at each residue, the energetic effect of the change was obtained by calculating the ΔΔG, the difference between the overall energy ΔG of each residue in the mutated sequence subtracted from the ΔG of residues of the unmutated 3D7 reference sequence as follows:
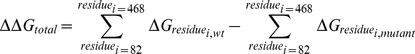



Briefly, MUMBO works by repacking amino acid side chains using the input structure backbone as a scaffold. The residues are built onto the scaffold using parameters derived from a standard library of crystal rotamer conformations for each amino acid. The energies for different rotamer combinations are assessed, and the energetically lowest is taken, which is consistent with the most stable packing. To overcome the problem of an exponentially expanding combinatoric space to explore, dead-end elimination is used to discard conformers and their combinations clearly producing energies far from the minimum, such as would arise from van der Waals clashes, thereby reducing the search space to a more tractable size. The force field used to compute the energy is the standard molecular mechanics atomistic potential energy function, as follows, using Chemistry at Harvard Macromolecular Mechanisms (CHARMM) parameters [32]:
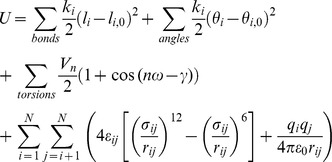



Thus, for each mutation, we rotamerized the mutation site, as well as all the wild type residues, such that the entire structure was repacked each time. The reference sequence from *P. falciparum* clone 3D7 (PF3D7_0304600) was also completely repacked to obtain the reference state ΔG, and the predicted protein structure obtained was similar to the published crystal structure [19]. The MUMBO analysis was first used to look for energetically constrained residues by determining positions where the average change in the Gibbs free energy (ΔΔG) upon mutation (conservative measurement) deviated substantially from identical residues elsewhere within the sequence (i.e., a given mutation exceeds two standard deviations from the average for the same residue at other positions within the reference structure) (Table S4).

We also examined the ΔΔG mutation profile for each position across all TH2 and TH3 residues from the perspective of the ancestral allele which was inferred from multiple alignments of *Plasmodium* species [19]. For positions where 3D7 residue was not the probable ancestral residue, the free energies were renormalized so that the putative ancestral residue ΔΔG was zero. Across all TH2 and TH3 residues the median free energy change from the ancestral residue was calculated at each position for all 19 possible non-ancestral residues. Observed residue polymorphisms were categorized as increased or decreased free energy compared to the median ΔΔG. In neutral non-functional sequence, the free energy will not impact the sequence and thus any amino acid may be equally likely to evolve as a polymorphism at a given position. In a sequence with conserved function, ΔΔG is usually minimized and thus the majority of changes would be expected to fall below the median. Conversely, outside forces such as intermolecular interactions or other external selective forces are usually required to elicit drastic changes in ΔΔG. To detect the likely effects of positive selection the observed categories were compared using a binomial distribution that models the neutral expectation of increases and decreases relative to the median being equally observed.

## Results

In Malawi, the 100 participants had an average multiplicity of infection (MOI) of 2.35 leading to 235 parasite variants being identified [5]. These represented 57 unique parasite haplotypes. In the Gambia, there were 25 unique haplotypes in 55 variants. Of these haplotypes, 13 TH2/TH3 haplotypes were shared between the two sites, representing 23% of Malawian and 50% of Gambian isolates. Individually, 13 TH2 types were shared and 10 TH3 types were shared (Table 1 and 2). Upstream from the TH2 and TH3 epitopes (amino acids <311), several polymorphic sites were also identified in both populations. Further analysis of these was precluded as these regions were not part of the available protein crystal structure. Variable amino acids upstream of TH2 were conserved between the sites with the exception of an E295K mutation found only in the Gambia population (Figure 1).

**Table 1:** Shared TH2 between Malawi and the Gambia.

Type	S	D	K	H	I	K	E	Y	L	N	K	I	Q	N	S	L
TH2-1	.	.	Q	.	.	E	K	.	.	K	I	.	.	.	.	.
TH2-2	.	.	Q	.	.	E	K	.	.	K	T	.	.	.	.	.
TH2-3	.	.	.	.	.	E	Q	.	.	K	T	.	.	.	.	.
TH2-6	.	.	Q	.	.	E	K	.	.	K	T	.	K	.	.	.
TH2-7	.	.	.	.	.	E	Q	.	.	K	T	.	K	.	.	.
TH2-8	.	.	.	.	.	E	Q	.	.	K	R	.	.	.	.	.
TH2-9	.	.	.	.	.	.	.	.	.	.	.	.	.	.	.	I
TH2-10	.	.	Q	.	.	E	K	.	.	K	R	.	.	.	.	.
TH2-23	.	.	.	.	.	E	Q	.	.	K	I	.	R	.	.	.
TH2-28	.	.	Q	.	.	E	K	.	.	K	.	.	.	.	.	I
TH2-29	.	.	Q	.	.	E	K	.	.	Q	.	.	.	.	.	.
TH2-30	.	.	Q	.	.	E	K	.	.	K	.	.	.	.	.	.
TH2-31	.	.	.	.	.	E	.	.	.	K	.	.	K	.	.	I

**Table 2:** Shared TH3 epitopes between Malawi and the Gambia.

Type	N	K	P	K	D	E	L	D	Y	A	N	D
TH3-0	.	.	.	.	.	.	.	.	.	.	.	.
TH3-1	.	.	.	.	.	Q	.	.	.	.	.	.
TH3-2	.	.	.	.	.	Q	.	.	.	E	.	.
TH3-3	.	.	.	.	.	.	.	N	.	E	.	.
TH3-4	D	.	.	.	.	Q	.	.	.	I	.	.
TH3-5	D	.	.	.	.	Q	.	.	.	.	.	.
TH3-6	.	.	.	.	.	Q	.	N	.	E	.	.
TH3-7	.	.	.	.	.	.	.	.	.	E	.	.
TH3-8	G	.	S	.	N	.	.	.	.	E	.	.
TH3-14	D	.	.	.	N	.	.	.	.	E	.	.

**Figure 2:**
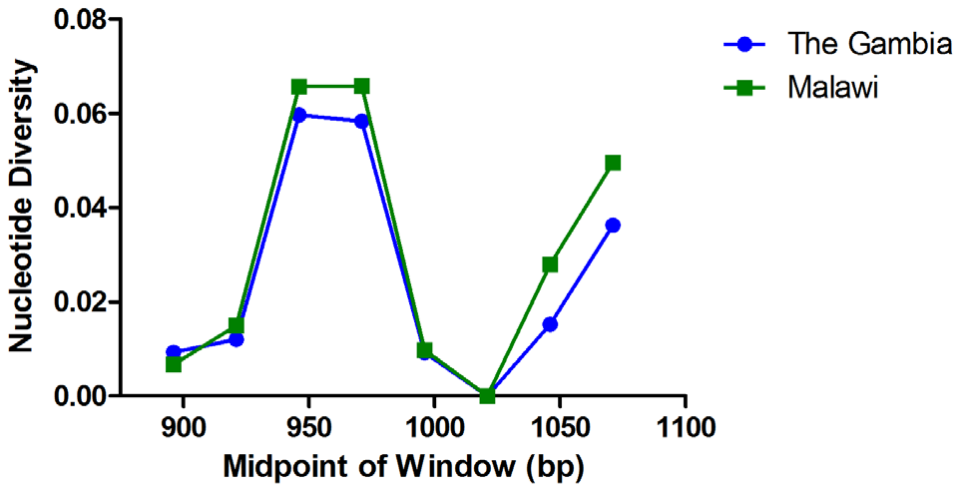
Nucleotide Diversity of *pfcsp* from Malawi and the Gambia. The nucleotide diversity (π) was determined for the sequenced region using a sliding window (size: 50 bp, slide: 25 bp). Two peaks in diversity are seen corresponding to the TH2 (bp 897–972) and TH3 (bp 1046–1091) epitopes. The peak level of diversity and pattern of diversity was similar between the two populations.

In order to assess the extent of genetic diversity and the extent of genetic similarity between populations, we investigated the nucleotide diversity of this 220 bp region of CS. In general, both populations had high levels of haplotype diversity (H_d_: Malawi = 0.957 and Gambia = 0.953), which essentially is the measure of two random strains within the population having different haplotypes. The average number of pairwise nucleotide differences expected between two strains (K) was similar (6.00 vs. 6.68) with similar overall nucleotide diversity (π) diversity (0.023 vs 0.025), which is K normalized for the length of the sequence. Measures of nucleotide diversity are summarized in Table 3. The level of nucleotide diversity across this region is known to be uneven; therefore we re-evaluated nucleotide diversity (π) for each population using a sliding window approach (50 bp size, 25 bp slide) across the T cell epitopes using the program DnaSP (Figure 2). As expected, the regions of peak nucleotide diversity correspond to the TH2 and TH3 epitope regions, with the maximum diversity seen between positions 897 to 972 (corresponding to the TH2 epitope). Interestingly, in both populations, all polymorphisms were nonsynonymous indicating that this region of the *pfcsp* gene is likely to be under strong selection. Since diversification of haplotypes within this region may also occur due to recombination, we estimated the minimum number of recombination sites using DnaSP v5.10.01 [23]. A high number of recombination sites were predicted in both populations (8 in Malawi and 7 in the Gambia). In Malawi, the majority of these (6) were located within the TH2 (nucleotides 978–1029) and TH3 (nucleotides 1100–1135) epitopes themselves, suggesting recombination may be important for generation of diversity in these sites (Table 3). A single recombination event was detected between the two epitopes. Between the two populations, the fixation index (F_ST_), a measure of the population differentiation due to genetic structure was 0.034 suggesting little genetic distance between the populations. We confirmed this using both phylogenetic and statistical methods. A Hudson’s Nearest Neighbor analysis, a test measuring how often the nearest neighbors are from the same population, showed no significant geographic separation of haplotypes (S_nn_ = 0.440; ns). A neighbor joining network was constructed in MEGA and visually shows no evidence of geographic clustering (Figure S2). These data suggest that the levels and distribution of nucleotide diversity are similar in Malawi and the Gambia, and that these two populations, separated by an extended geographic distance, are remarkably genetically similar at the nucleotide level.

**Table 3:** Recombination, Diversity, and Genetic Distance for Malawian and Gambian Parasites.

	Malawi (n = 235)	Gambia (n = 55)	
**Recombination**			
Rm (Minimum number of recombination events)	8	7	
Singleton variable sites (nt position)	2 (1007, 1017)	3 (947, 951, 1017)	
Parsimony Informative sites (nt position)	22 (940, 942, 948, 986, 995, 996, 1004, 1009, 1010, 1016, 1025, 1100,1101, 1106, 1112, 1114, 1115,1121, 1127)	11 (895, 941, 950, 962, 964, 971, 980, 1055, 1030, 1070, 1076)	
No. Synonymous mutations	0	0	
No. Non-synonymous mutations	20	20	
Recombination sitesTH2: 977–1027TH3: 1100–1135	[948,986] [995,1009] [1009,1010] [1010,1016] [1016,1025] [1025,1100] [1106,1112] [1115,1121]	[895,950] [950,964] [964,971] [971,980] [980,1055] [1055,1070] [1070,1076]	
**Diversity**			
Hd (Haplotype diversity) +/− SD	0.957±0.005	0.953±0.016	
K (Average number of pairwise nucleotide differences)	6.003	6.682	
π (Nucleotide diversity) +/− SD	0.023±0.001	0.025±0.002	

**Figure 3:**
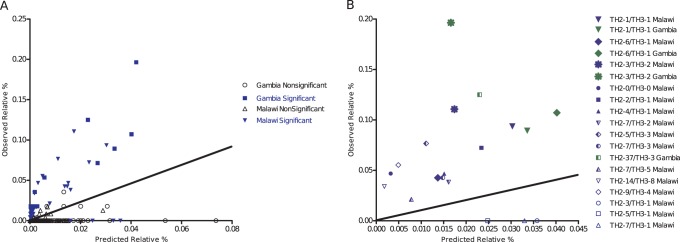
TH2 and TH3 Pairings from Malawi and the Gambia. (a) This figure shows all TH2-x/TH3-y haplotype pairings comparing observed and expected. Those that are that are statistically over and under represented based upon our contingency table analysis (p≤0.00009 for Malawi and p≤0.003 for the Gambia) are colored blue. (b) The data shown are for those pairings in Malawi (blue) and the Gambia (green) that are either observed >5 times in our data, or those predicted to occur >5 times based upon our contingency analysis. Each pairing is represented a unique symbol. Of note, three pairings (TH2-1/TH3-1, TH2-6/TH3-1, and TH2-3/TH3-2) were over represented in both populations. In both figures, the diagonal line represents if there were non-random association of pairings based on predicted and observed values. Points above the line represent pairing over represented in the population, while those represented below the line are those under represented in the population. A complete list of these significant pairings is provided in Table S2 and S3.

Previous reports have suggested that the association between TH2 and TH3 epitopes is not random [1]. We assessed the distribution of the specific epitopes of TH2 and TH3 among the 235 Malawian and 55 Gambian variants identified. We used simulations to test whether the TH2 and TH3 epitopes were randomly associated within the sequence haplotypes. Based on these simulations, a model of random associations was rejected in Malawi [p<0.001, Degrees of freedom (df) = 490, G2 statistic = 824]. Among the Gambian isolates, we did not see a statistically significant overall departure from the null model of random assortment for the entire population [p = 0.298, df = 171, G2 statistic = 180.3]; perhaps secondary to the lower statistical power due to limited number of isolates. However, there was significant over-representation of certain combinations. If the assortment of T cell epitopes were non-random, we would expect the observed frequencies to be equal to the predicted frequencies from the contingency table analysis. Instead, we see many pairings in which the observed frequency is significantly higher or lower than the predicted frequency of a pairing (Figure 3). The complete contingency table is shown in Table S1 and the list of all statistically significant pairing is shown in Table S2 and Table S3. This suggests that similar to what was seen in Kenya, the associations between TH2 and TH3 are not random and the possible combinations that occur within natural populations are constrained by biology and/or by limited time for recombination to randomly reassort the mutations.

**Figure 4:**
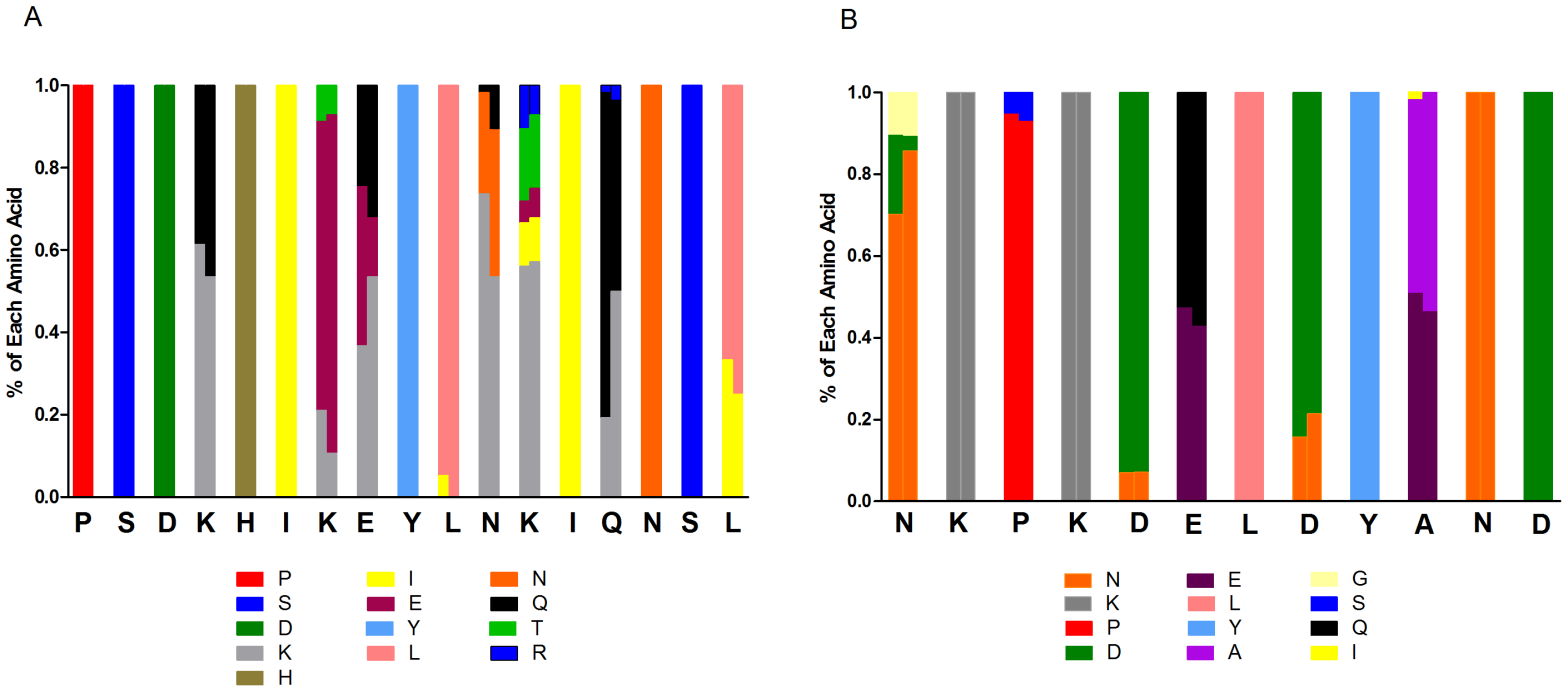
Amino Acid Distribution in TH2 and TH3. The distributions of amino acids in the TH2 (Panel A) and TH3 (Panel B) epitopes are shown. The reference 3d7 amino acid sequences are shown on the X axis. Above the amino acid is a column representing the frequency of amino acids seen in isolates from Malawi (left half of the column) and the Gambia (right half of the column). The relative frequency of each amino acid is similar between the two geographically-distant African populations.

Patterns of amino acid polymorphisms within the epitopes were then assessed. The sequence logo of the amino acid sequence for both countries (Figure 1) suggests positional bias in the diversity of CS. There were no significant differences between the two populations, with the exception that position 295 is variable in the Gambia, but monomorphic among the Malawian isolates. Within the TH2 and TH3 epitopes, the distribution and frequency of amino acid type were evaluated (Figure 4) showing highly similar amino acid polymorphisms, with similar frequencies, between the populations. Interestingly, within Malawi, we found 118 (50.2%) variants having at least a TH2 or TH3 epitope within one amino acid of the 3D7 (RTS,S vaccine) epitopes, while 230 (97.9%) have a TH2 or TH3 epitope within two amino acids of 3D7 epitopes. Using the sequence logo, we identified ten sites within the TSP domain which are most highly mutable, namely positions 314, 317, 318, 321, 322, 324, 327, 352, 357, and 361 (information content ≤3). These polymorphic sites predominately involve positively or negatively charged residues. Interestingly, positions 314, 317, 318, 321 and 324 can be populated by either positive or negative residues, suggesting that any charge-charge interactions are poorly conserved.

**Figure 5:**
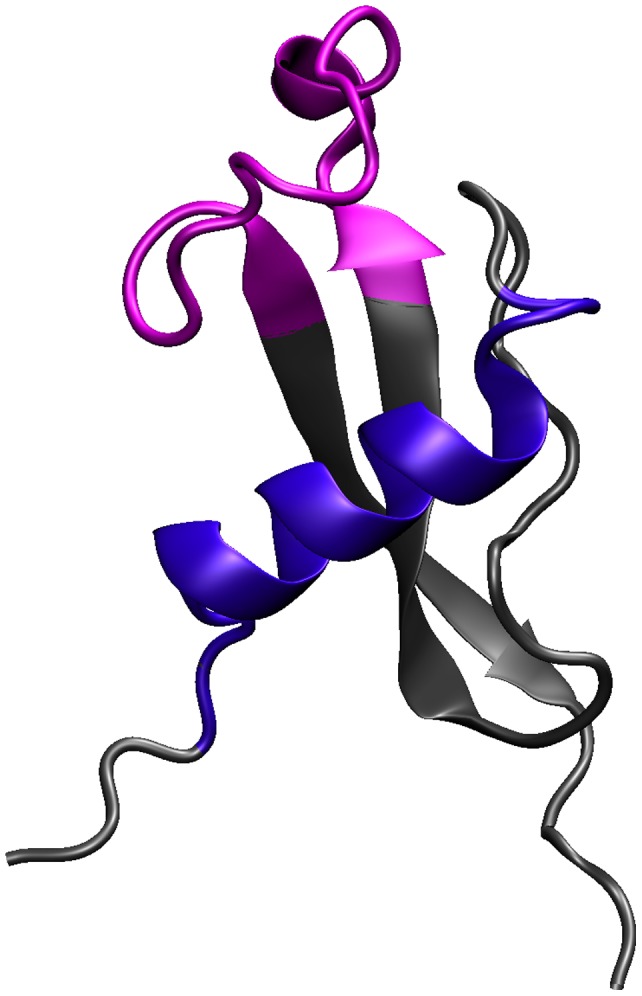
Location of TH2 and TH3 epitopes in the Structure of Circumsporozoite Protein. The relative location of TH2 (blue) and TH3 (pink) are shown within the protein structure.

**Figure 6:**
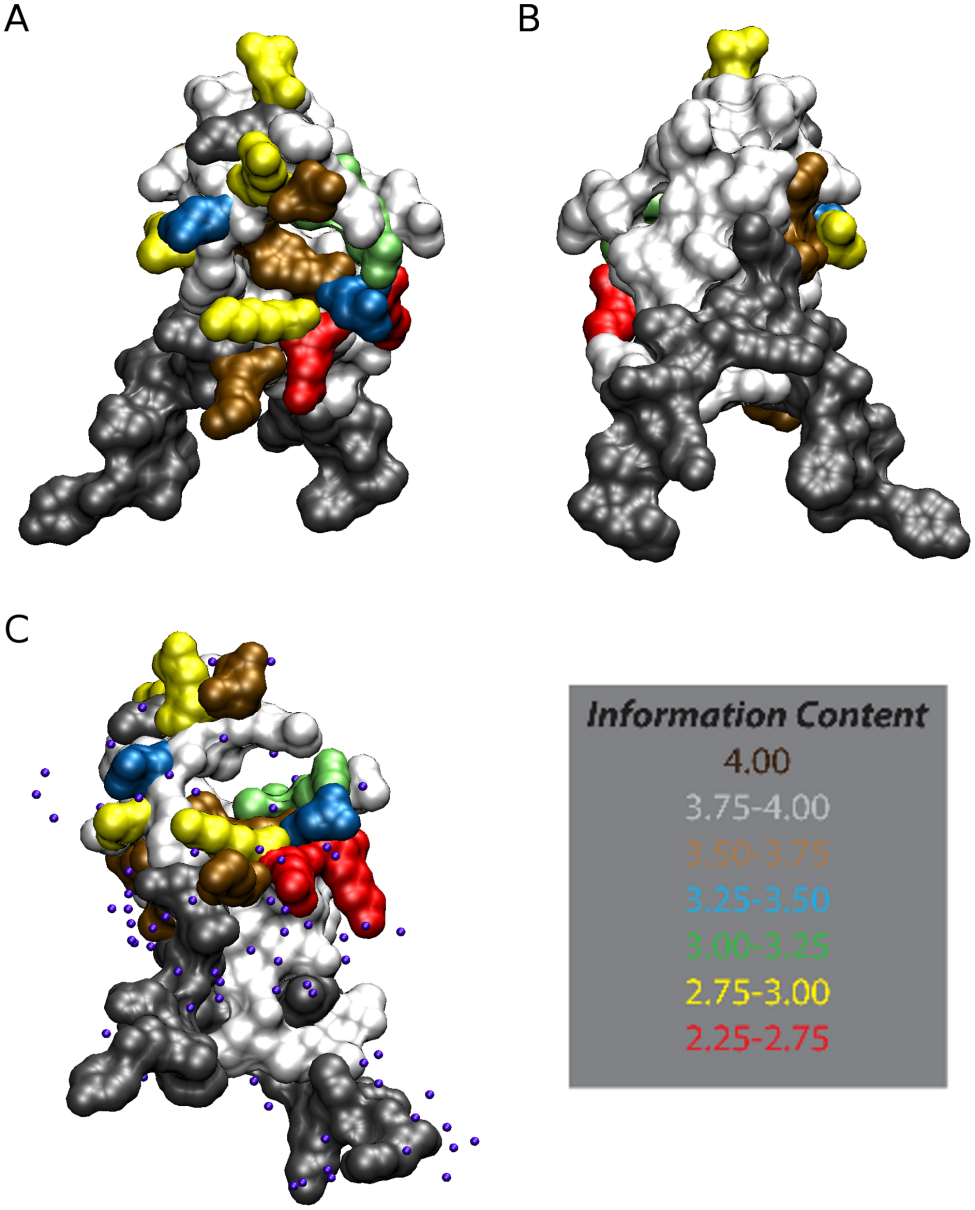
Sites of Polymorphism Mapped to the CS structure. The variable amino acids within TH2 and TH3 are shown in Frontal (Panel A), Back (Panel B) and Pocket (Panel C) views. Panel C includes solvent as spheres. Based upon the sequence logo for Malawi, the information content (IC) in bits binned at increments of 0.25 was mapped to the crystal structures via a color scheme indicating the magnitude. The information content is a relative measurement of sequence conservation, with higher values being more highly conserved and lower values having more diversity at that sequence position. This value is based upon the R_seq,_ which is determined by the difference between the maximum possible entropy and the entropy observed in the distribution at that location [30]. The maximum sequence conservation per site is dependent on the number of distinct symbols possible (20 for amino acids) and is therefore 4.32 bits for protein sequences. In this figure, red residues (IC<2.75) are the most highly mutable, followed by yellow (IC <3.0). The majority of residues that are highly variable face the external matrix and are not associated with the back of the molecule or included within the pocket.

Using the recently published crystal structure PDBID 3VDJ, we sought to conduct structural mapping of the highly mutable sites to gain insight as to how they are spatially oriented and related to one another. A surprising feature of the 3VDJ structure is its lack of resemblance to homologous domains in proteins such as thrombospondin, f-spondin and ADAMTS13, which have two antiparallel β sheets and one additional antiparallel strand, all held together by disulfide bridges [19]. The CS structure, on the other hand, features a short α-helical portion capped by a loop that folds onto the structure, and the N-terminal strand is ordered into an α-helix tethered beneath the flap by a hydrophobic stacking interaction of Trp 331 into the antiparallel β sheets. The highly mutable sites map to the αhelix, formed by the TH2 epitope, and the flap, formed by the TH3 epitope (Figure 5). Furthermore, the novel pocket created by this unusual structure is comprised of highly conserved residues. The most polymorphic residues point away from the pocket. The surface views, rendered with a probe having a radius of 1.4 angstroms, the size of a water molecule (Figure 6), show that the conserved pocket is quite large and readily accessible to solvent. Furthermore, the rear surface of the structure is highly conserved.

Similarly, we examined the structural mapping of those combinations of epitopes that were identified as significantly over represented in our analysis of TH2 and TH3 association (Table S2 and S3). Examination of the pattern of polymorphism within and between TH2 and TH3 epitopes did not reveal any patterns consistent with spatial interaction (compensatory mutations) suggestive of intramolecular interactions within an epitope or between epitopes. This may suggest that the interactions underlying the selection of the observed polymorphisms are entirely due to intermolecular rather than due to functional structural limitation. Given the disruptive nature of the amino acid changes predominantly facing one side of the protein this would be consistent with a diversifying pattern of intermolecular interaction of the intact protein consistent with immune evasion (e.g. disruptive binding to epitope specific antibodies rather than HLA-binding peptide epitopes) or co-evolution with a host receptor.

**Figure 7:**
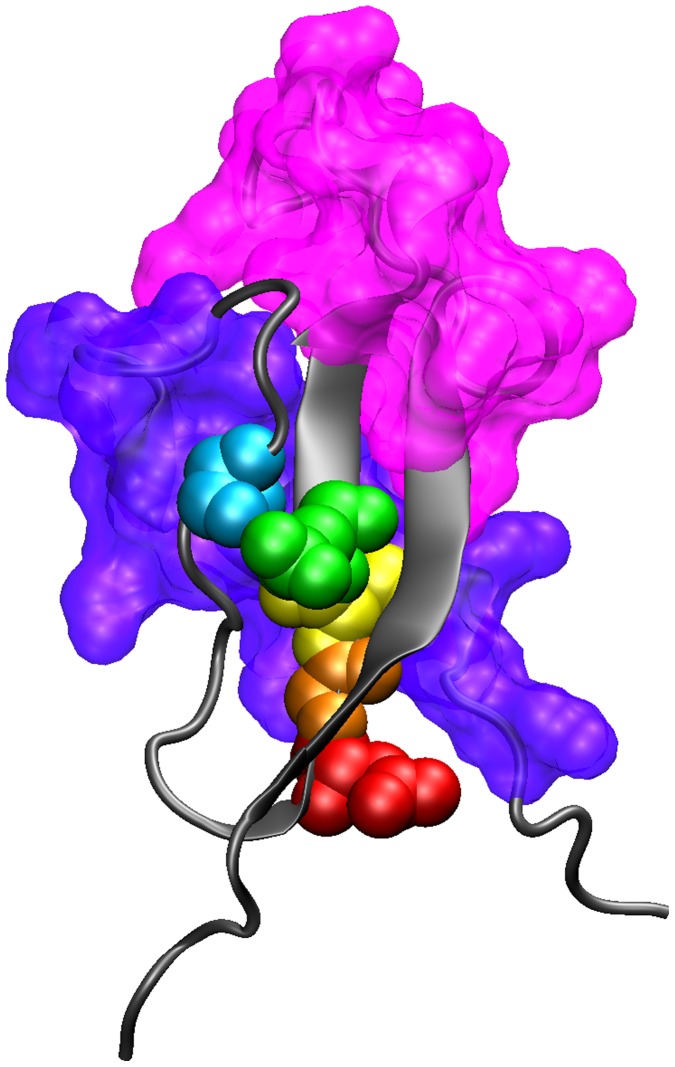
Significantly energetically-constrained amino acid positions identified by MUMBO Analysis. The 5 amino acids identified by MUMBO as having constrained ΔΔG mutational profiles relative to other identical amino acids within the crystal structure are color coded: ASN340 (red), Gly341 (orange) Ile342 (yellow) Glu343 (green), and Ser332 (cyan), are shown with respect to the TH2 and TH3 domains’ surface area (colored as in Figure 4). These residues cluster behind the conserved hydrophobic pocket and were identified because there mutational profile differed on average by 2 standard deviations from all other identical residues within the crystal structure.

Calculation of Gibbs free energies on exhaustively mutagenized structures can provide information on structural constraints of a protein. Given the newly evolved fold-flap and pocket in the *P. falciparum* CS, polymorphic changes could reflect a lack of structural constraint in this region. To study the energetic constraints and effects of point mutations, we performed a comprehensive point mutation analysis of the structure using MUMBO software. This yields an estimate of Gibbs free energy required for each of the possible alternate states, indicating the favorability of making each of the 19 residue substitutions theoretically possible at each position in the reference sequence (Table S4). After quality control checks to validate the appropriateness of the method to the CS structure, we searched for residues which behaved anomalously (differing by at least 2 standard deviations) when changed from the reference state relative to similar residues at other positions. Five such residues were identified. Substitution of Asn 340 with Leu, Ile, and Val was predicted to be particularly favorable on the grounds of energetics (Figure S3), suggesting that mutation towards an aliphatic residue from a negatively charged residue was highly permissible. Substitution of Gln 343 to the aromatics residues His, Tyr, Trp, and Phe was strongly disfavored in this analysis and a similar trend was observed for Ser 332 and Ile 342. Substitution of Gly 341 by any other amino acid generates a substantial energy increase. This is supported by comparison between species of malaria, in which Gly 341 is conserved among all species, while the other residues have one alternate state (S332T, N340V, I342V, and Q343R) [19]. Upon mapping these residues to the structure (Figure 7), they clustered behind the conserved hydrophobic pocket, falling on β strand 2 except Ser332, which packs with β2. The observed tight packaging within the structure probably causes spatial constraints, disfavoring the incorporation of large amino acid substitutions. Gly appears to be selected for its small size, given the large van der Waals forces likely to be generated by substitutions from this smallest amino acid. The location of these restricted residues in relation to the pocket suggests that both the pocket and the packed core need to be highly conserved for stabilization of the molecule.

The mutagenesis studies can also be used to identify sites likely to be under selection by applying inductive reasoning. The presence of polymorphisms in a protein can be due to a lack of evolutionary constraint and/or selective pressures leading to diversification. Beginning with the supposition that in lieu of other forces or constraints (e.g. functional or immune interactions) a protein will evolve towards a more stable confirmation one might expect an excess of energetically favorable residues and polymorphisms arising over time. However, if an energetically unfavorable residue were to populate a position more frequently than expected, an intramolecular or intermolecular selective pressure may be acting on the sites of mutation or polymorphism. We examined the relative energetics of all 19 mutational possibilities at each site across TH2 and TH3. If intramolecular interactions determine the sites of polymorphism then polymorphic sites could be expected to have lower median ΔΔGs on average reflecting less constraint (i.e. a greater subset of energetically accessible/reasonable residues). Initial comparison revealed a slight but insignificant difference in median ΔΔGs between fixed and polymorphic positions (average: 3.14 vs. 1.41 respectively, p = 0.43, t-test). Upon excluding the hydrophobic sites, which are highly constrained, the difference between the median ΔΔGs of the fixed and polymorphic sites decreased (average −0.86 and −0.39. respectively p = 0.60, t-test). This suggests that simple intramolecular energy constraints are not appreciably determining the pattern of polymorphism within TH2 and TH3.

**Figure 8:**
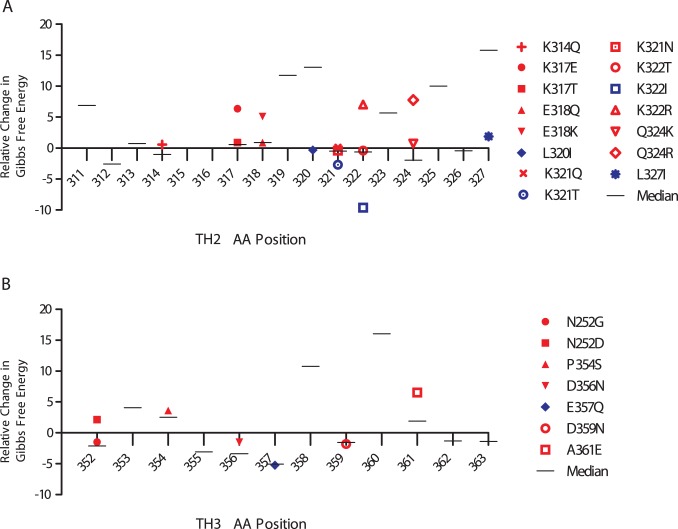
Calculated ΔΔG of observed polymorphic amino mutations from the ancestral amino acid residue compared relative to median of all possible mutations at each position. Free energy changes of polymorphisms in TH2 and TH3 are shown relative to the median change from all 19 substitutions from the predicted ancestral allele determined from *Plasmodium sp.* phylogeny. Mutations that have higher energy than the median are shown in red, while those with lower energy are shown in blue. Positive values represent increases in free energy and thermodynamic instability while negative values represent decline in free energy and greater stability. Neutral sequence where energetics have no effect would be expected to occur 50/50 above and below the median, while conservation of intramolecular function would be expected to minimize entropy and lead to lower energy states. Intermolecular interactions can lead to selection for less favorable states which are significantly enriched in the observed polymorphisms (17 increased vs 5 decreased, p = 0.00845).

To determine if intermolecular forces play a role in shaping the diversifying polymorphisms in TH2 and TH3, we devised a simple and conservative test for intermolecular forces. If there are no intermolecular selective forces acting on a site then we expect that observed mutations will be energetically more favorable and increase protein stability. In the worse case, a protein may be under no constraints and essentially adrift with random residue changes occurring regardless of energetics. In this case we would expect that observed residue changes would be equally likely to be greater than or less than the median ΔΔG at a given position. Thus, we would expect a 50/50 neutral model if we aggregated across TH2 and TH3. However, we observe 17 polymorphisms with ΔΔG greater than the median and only 5 less than the median (p = 0.00845, exact binomial distribution) from the predicted ancestral state (Figure 8). For example position 317 contains Lys and Glu whereas favorable energetic mutations to Leu, Ile, Val, Tyr, Trp, and Phe do not substantively populate position 317. Similarly, the dominant mutations 318 Glu/Gln/Lys, 321 Gln/Lys, 322 Lys/Thr, 324 Gln/Lys, and 361 Gly/Glu all have more energetically favorable options which do not appreciably manifest themselves. Given this relative unfavorability within the context of the protein compared to our conservative neutral model (presuming this protein has no energetic constraints), this suggests that outside intermolecular selective pressures, either immunological or functional (e.g. receptor binding), have shaped the pattern and nature of the TH2 and TH3 polymorphisms.

## Discussion

In this study, we describe polymorphism in the *P. falciparum* gene *pfcsp* in two natural parasite populations and map predicted amino acid substitutions onto the recently elucidated crystal structure of the C terminal end of the CS protein. Our analysis then investigates how these polymorphisms might impact intramolecular interactions and may be shaped by intermolecular interactions. Such analyses are important for several reasons. First, concerns have been raised about the impact of antigen diversity on the development of effective malaria vaccines. Recent studies have suggested that diversity in vaccine targets can seriously compromise efficacy, as a recently tested apical membrane antigen 1 (AMA-1) vaccine was found to be efficacious only against those parasites with AMA-1 alleles similar to the variant in the vaccine construct [33], [34]. Previous work has been contradictory regarding selection in this region. Some studies have suggested that the selective pressures put on CS by naturally acquired immunity and vaccine induced immunity appear to be modest [1], [6], [13], [35]. On the other hand, this region has an extreme skew of nonsynonymous polymorphisms compared to synonymous polymorphisms [5], [8], which suggests there is selection leading to the diversification of this region. Even weak or modest selection has the potential to affect the long term utility of a vaccine by promoting selection of vaccine resistant strains. Analysis of *pfcsp* variants in breakthrough infections in RTS,S vaccine recipients have not shown strain selection, but these were in Phase II trials, and may have been underpowered to detect all but the strongest effect [1], [6], [35]. Second, the variation can shed light on the biological role of the protein. Having the empirically determined crystal structure elucidated for a malaria vaccine antigen provides an opportunity to investigate the interplay among sequence variation, structure/function requirements, and host immune selection in natural parasite populations. The integration of structural analysis with clinical response to infection has previously been used in evaluating the impacts of diversity on P. falciparum apical membrane antigen 1 (*pfama1*) [4].

We chose two well-separated parasite populations for our analysis on the understanding that inter-population differences, as well as intra-population sequence diversity, would be informative. However, the overall sequence diversity in *pfcsp* was similar in both populations (Table 1 and 2, Figure 1), did not differ from that observed in previous studies in Africa [13], [18], [36] and showed very little genetic differentiation between the two populations (Table 3 and Figure 4). This suggests that genetic drift may not be an important source of variation at this locus, and that the results presented from the two parasite populations in the study are likely generalizable to much of Africa [18].

In a previous study in Kenya, Waitumbi *et al* [1] found evidence that associations between TH2 and TH3 epitopes were non-random, leading to the suggestion that there are functional limitations on polymorphism in this region of the gene. However, in that study only the most common *pfcsp* alleles were assessed. Our study confirms this finding in two additional African cohorts, showing that within a population, certain TH2 and TH3 combinations appear to be over- and under-represented. There are several potential explanations for this phenomenon. First, as suggested by these authors [1], this may represent structure/function limitations that restricts the potential combinations of T cell epitopes within a CS variant. Our structural studies do indicate that the TH2 and TH3 regions are in close proximity; however, the most polymorphic residues are solvent-exposed and lack correlated mutations approximating each other in physical space, suggesting that direct interactions are unlikely (provided that this is the biologically active conformation of the molecule). The analysis did not reveal any patterns consistent with spatial interaction (compensatory mutations) allowing for intramolecular interactions within an epitope or between epitopes. This suggests that all of the interactions underlying the selection of this polymorphism are intermolecular rather than due to functional structural limitation. Such intermolecular interactions may even include CS-CS associations in the formation of the sporozoite coat [37]–[39]. Second, these combinations may represent the impact of a selective force limiting the distribution of haplotypes. Assessment of the complete population of parasites from Lilongwe, Malawi (Table S1) shows that while some combinations are clearly dominant (e.g., 22 of TH2-1/TH3-1 type), there is a plethora of uncommon variants that are still circulating with a wider distribution of TH2 and TH3 linkages. This may suggest that recombination may randomly generate diversity through this region, but selective limitations placed on the parasite population prevent the enrichment of certain pairings. This selection could potentially be driven by the overall immunity to specific variants that may fluctuate over time or potentially even occur within the mosquito vector. Third, it is not surprising that a strong association between TH types may exist due to the close physical proximity of the two epitopes within CS.

The patterns of amino acid substitutions in the TSR domain of CS were assessed in the two geographically-distant populations, showing that the most mutable amino acids (information content <3.0 on the sequence logo) were confined to the TH2 and TH3 epitopes. The frequency of mutated amino acids in each position was similar between the different populations, suggesting that certain polymorphisms are preferred (Figure 4). These polymorphic sites are highly correlated with those seen in Kenya and Peru [1], [40].

The extensive mutagenesis analysis of TH2 and TH3 detected no significant differences between the fixed and polymorphic positions. Additionally, our analysis of the energetics suggests that there are a disproportionate number of energetically unfavorable polymorphisms implying that intermolecular forces are acting to select for such changes (Figure 8). Such intermolecular interactions are more likely at the protein surface, supported by the fact that these polymorphisms are confined exclusively to the front of the molecule, surrounding, but not involving, the pocket (Figure 6). The extensive analysis of simulated mutagenesis clearly suggested that residues surrounding and behind the pocket are not permitted to vary and have specific energy requirements (Figure 7), denoting that this region must remain conserved likely for functional or structural reasons. Thus the scenario could be that functionally important sites requiring specific amino acids, such as the pocket itself and residues closely packed against it, are conserved to maintain binding to a conserved host receptor, whereas surface sites allow mutagenesis to evade host immune recognition that might interfere with the function of the pocket. Charge reversals would indicate relaxed constraints only needing to meet the criteria of being able to engage in polar interactions. Mutating to a different charge meets that criterion, while making the site more difficult to be recognized by a residue specific interaction such as may occur in the immune response and affords strains a new charge as a means of evading the immune response while retaining functionality. So long as this unfavorable mutation is not sufficient to cause structural perturbation of requisite function, the evolutionary advantage would outweigh the energetic penalty, thereby driving protein evolution energetically uphill and being selected by evading the immune response. Conversely, the observed polymorphisms could be due to diversification of a portion of the binding ligand combined with conservation of an important functional element with which the pocket interacts. Given the structural co-localization of TH2 and TH3 around the hydrophobic pocket, diversification due to processed peptides and MHC binding dependent only on primary structure now appears less parsimonious.

Given the disruptive nature of the amino acid changes, it is possible that the polymorphisms themselves so alter the secondary and/or tertiary structure of the CS protein that the crystallography data generated from the 3D7 variant are not a valid scaffold for structural mapping of variant epitope combinations. This could be tested by crystallographic analysis of a variant of CS dissimilar to that of 3D7. Furthermore, both the crystal structure and our predictive structural mapping were performed with no reference whatsoever to the NANP repeats which comprise the bulk of the amino-terminal half of the CS molecule and can be highly variable in number. However, the fact that the C terminus is so tightly folded suggests that it has a solid hydrophobic core and would likely be resistant to structural changes observed or due to distal effects in the NANP repeat.

Taken together, our results suggest that the patterns of diversity within the T cell epitopes within the TSR domain of CS are in part determined by the relative location of polymorphic amino acids within the intact protein structure. While our data argues that inter-molecular interactions of the intact protein are likely key to the observed diversity, it does not exclude a role for the T cell responses that have been observed in exposed individuals. However, it does raise the possibility that T cell responses are not the primary driver of polymorphism. Given that the TSR domain is well conserved across species and found 187 times within the human proteome [19], the T cell responses observed may in large part be due to the divergent nature of the TH2 and TH3 region being recognized as non-self by the human host’s immune system. Thus, any functional impact that these regions have in driving the T cell response may be a consequence of the diversity rather than the cause of the diversity. In any respect, given the limitations of our *in silico* analysis, this calls for renewed and broader empirical work to elucidate the selective forces driving the diversity in the TH2 and TH3 regions. This should include the evaluation of the potential strain specificity of antibody-mediated immune responses to these epitopes and a better understanding of the impact of vector biology on selection of parasite variants. Such experiments are required to fully understand the potential impact of large-scale vaccination and to truly optimize vaccine design to CS.

## Supporting Information

Figure S1 **Example of the Determination of TH2 and TH3 Pairing From a Parasite Haplotype.** This figure shows how each T cell epitope was coded into a unique type and how each pairing was determined. Red characters represent polymorphisms differing from the reference sequence. The unique TH2 types became rows on the contingency table (Table S1) while the unique TH3 types became columns on the contingency table. Each parasite isolate/sequence in the population was coded in a similar manner. This results in each cell in the contingency table being populated by the frequency of each unique TH2 and TH3 pairing.
(EPS)

Figure S2 **Neighbor-Joining (NJ) Phylogenetic Tree of** ***pfcsp*** **from Malawian and Gambian Isolates.** This figure shows the NJ tree for the 220bp fragment analyzed for the 57 unique haplotypes from Malawi (filled circles) and the 25 unique haplotypes from the Gambia (empty diamonds). Major branch divisions were estimated by bootstrapping 500 replicates. As suggested from the population genetic statistics, no population structure based upon geographic origin of haplotypes can be inferred. The NJ tree was created using MEGA software, Version 5.
(EPS)

Figure S3 **MUMBO Analysis.** This figure shows the MUMBO analysis of the five residues with special energy requirements. Panel A represents the energy requirements for SER332. Panel B, C, D and E show them for GLY341, ILE342, GLN343, and ASN340 respectively. The Y axis represents the ΔΔ Gibbs Free energy while the potential amino acids are on the X-axis.
(EPS)

Table S1 **Observed Frequencies of TH2-TH3 haplotype pairings within Malawian and the Gambian populations.**
(DOC)

Table S2 **List of significant (p≤0.00009) pairings of TH2 and TH3 Epitopes among Malawian Parasite Isolates.**
(DOC)

Table S3 **List of significant (p≤0.003) haplotype pairings of TH2 and TH3 Epitopes among Gambian Parasite Isolates.**
(DOC)

Table S4 **Average and Standard Deviation of Free Energy of Mutation.**
(DOC)
