# Supplementary material for: Diversity of T Cell Epitopes in Plasmodium falciparum Circumsporozoite Protein Likely Due to Protein-Protein Interactions
Source: PLoS One. 2013 May 7;8(5):e62427. doi: 10.1371/journal.pone.0062427 (PMC3646838; doi:10.1371/journal.pone.0062427)
Supplement: Table S1 — Observed Frequencies of TH2-TH3 haplotype pairings within Malawian and the Gambian populations. (DOC) [file pone.0062427.s004.doc]

**Table S1. Observed Frequencies of TH2-TH3 haplotype pairings within Malawian and the Gambian populations.** Significant frequencies divergent from expected are colored green and blue for the respective populations. (M=Malawi and G=Gambia, blank cells represent zero occurrences)

|  | TH3-0 NKPKDELDYEND | TH3-1 .....Q...... | TH3-2 .....Q...E.. | TH3-3 .......N.E.. | TH3-4 D....Q...I.. | TH3-5 D....Q...... | TH3-6 .....Q.N.E.. | TH3-7 .........E.. | TH3-8 G.S.N....E.. | TH3-9 G........... | TH3-10 G...N....E.. | TH3-11 G...E..N.E.. | TH3-12 G........E.. | TH3-13 D........E.. | TH3-14 D...N....E.. |
| --- | --- | --- | --- | --- | --- | --- | --- | --- | --- | --- | --- | --- | --- | --- | --- |
| TH2-0 PSDKHIKEYLNKIQNSL | M11 |  |  |  |  |  |  |  |  |  |  |  |  |  |  |
| TH2-1 ...Q..EK..KI..... |  | M22 |  |  |  |  |  |  |  |  |  |  |  |  |  |
|  |  | G5 |  |  |  |  |  |  |  |  |  |  |  |  |  |
| TH2-2 ...Q..EK..KT..... |  | M17 |  |  |  |  |  |  |  |  |  |  |  |  |  |
|  |  | G4 |  |  |  |  |  |  |  |  |  |  |  |  |  |
| TH2-3 ......EQ..KT..... |  | M0 | M26 | M0 |  |  |  |  |  |  |  |  |  |  |  |
|  |  |  | G11 |  |  |  |  |  |  |  |  |  |  |  |  |
| TH2-4 ...Q..EK..K...... |  | M11 |  |  |  |  |  |  |  |  |  |  |  |  |  |
|  |  | G6 |  |  |  |  |  |  |  |  |  |  |  |  |  |
| TH2-5 ......EQ........I |  | M0 |  | M18 |  |  |  |  |  |  |  |  |  |  |  |
| TH2-6 ...Q..EK..KT.K... |  | M10 |  |  |  |  |  |  |  |  |  |  |  |  |  |
| TH2-7 ......EQ..KT.K... |  | M0 | M9 | M10 |  | M5 |  |  |  |  |  |  |  |  |  |
|  |  |  |  | G1 |  |  |  |  |  |  |  |  |  |  |  |
| TH2-8 ......EQ..KR..... | M4 |  |  |  |  | M4 |  |  |  |  |  |  |  |  |  |
|  | G2 |  |  |  |  |  |  |  |  |  |  |  |  |  |  |
| TH2-9 ................I | M1 | M3 |  |  | M13 |  |  | M2 |  |  | M2 |  |  |  |  |
|  | G1 | G1 |  |  | G1 |  |  |  |  |  |  |  |  |  |  |
| TH2-10 ...Q..EK..KR..... |  | M4 |  |  |  | M1 |  |  |  |  |  |  |  |  |  |
|  |  | G2 |  |  |  |  |  |  |  |  |  |  |  |  |  |
| TH2-11 ...Q..EK.....K... |  | M3 |  |  |  |  |  |  |  |  |  |  |  |  |  |
| TH2-12 ......K...K...... |  |  |  |  |  |  | M2 |  |  |  |  |  |  |  |  |
| TH2-13 ......EQ..K.....I |  |  |  | M1 |  |  |  | M3 |  |  |  |  |  |  |  |
| TH2-14 ......T...K...... |  |  |  |  |  |  |  |  | M8 |  |  |  | M1 |  |  |
| TH2-15 ......T...K..K..I |  |  |  |  |  |  |  | M3 |  |  |  |  |  |  |  |
| TH2-16 ...........E....I |  |  |  |  |  |  |  | M2 |  |  |  |  |  |  |  |
| TH2-17 .............K..I |  |  |  |  |  |  |  | M3 |  |  |  |  |  |  |  |
| TH2-18 ......EQ..KI.K... |  |  |  |  |  | M4 |  |  |  |  |  |  |  |  |  |
| TH2-19 ......T...K.....I |  |  |  |  |  |  |  | M2 |  |  |  |  |  |  |  |
| TH2-20 ......EK..KE..... |  |  | M2 |  |  |  |  |  |  |  |  |  |  |  |  |
| TH2-21 ...........E.K..I |  |  |  |  |  |  |  | M1 |  |  |  |  |  |  |  |
| TH2-22 ...Q..E...KT..... |  | M1 |  |  |  |  |  |  |  |  |  |  |  |  |  |
| TH2-23 ......EQ..KI.R... |  |  |  |  |  | M1 |  |  |  |  |  |  |  |  |  |
|  |  |  |  |  |  | G1 |  |  |  |  |  |  |  |  |  |
| TH2-24 ...Q..EK.IK...... |  | M2 |  | M2 |  |  |  |  |  |  |  |  |  | M1 | M1 |
| TH2-25 ......T...KR..... |  |  |  |  |  |  |  |  | M3 |  |  |  |  |  |  |
| TH2-26 ...Q..EK......... |  | M1 |  |  |  |  |  |  |  |  |  |  |  |  |  |
| TH2-27 ..........KI..... |  |  |  |  |  |  |  |  |  | M3 |  | M2 |  |  |  |
| TH2-28 ...Q..EK..K.....I |  |  |  |  |  |  |  | M1 |  |  |  |  |  |  |  |
|  |  |  |  |  |  |  |  | G1 |  |  |  |  |  |  |  |
| TH2-29 ...Q..EK..Q...... |  | G1 |  |  |  |  | M1 |  |  |  |  |  |  |  |  |
|  |  |  |  |  |  |  | G1 |  |  |  |  |  |  |  |  |
| TH2-30 ...Q..EK..K...... |  | G1 |  |  |  | M3 |  |  |  |  |  |  |  |  |  |
| TH2-31 ......E...K..K..I |  |  |  | M1 |  |  |  | G1 |  |  |  |  |  |  |  |
| TH2-32 ......E...K.....I |  |  |  | M1 |  |  |  |  |  |  |  |  |  |  |  |
| TH2-33 ......EK..KI..... |  |  |  | M1 |  |  |  |  |  |  |  |  |  |  |  |
| TH2-34 ...Q..E...K...... |  | M1 |  |  |  |  |  |  |  |  |  |  |  |  |  |
| TH2-35 ...Q..EK........I |  | M1 |  |  |  |  |  |  |  |  |  |  |  |  |  |
| TH2-36 ......EQ..K..K..I |  |  |  |  |  |  |  | G3 |  |  |  |  |  |  |  |
| TH2-37 ......EQ.....K..I |  |  | G1 | G7 |  |  |  |  |  |  |  |  |  |  |  |
| TH2-38 ...Q..EK..KI.K... |  | G1 |  |  |  |  |  |  |  |  |  |  |  |  |  |
| TH2-39 ..........T...... |  |  |  | G1 |  |  |  |  |  |  |  |  |  |  |  |
| TH2-40 .......K..KE..... |  |  |  |  |  |  |  |  |  |  |  |  |  |  | G1 |
| TH2-41 ......T...KR..... |  |  |  |  |  |  |  |  | G1 |  |  |  |  |  |  |
| TH2-42 ...Q..EK..K..K..I |  |  |  |  |  |  |  | G1 |  |  |  |  |  |  |  |
