# Supplementary material for: Diversity of T Cell Epitopes in Plasmodium falciparum Circumsporozoite Protein Likely Due to Protein-Protein Interactions
Source: PLoS One. 2013 May 7;8(5):e62427. doi: 10.1371/journal.pone.0062427 (PMC3646838; doi:10.1371/journal.pone.0062427)
Supplement: Table S2 — List of significant (p≤0.00009) pairings of TH2 and TH3 Epitopes among Malawian Parasite Isolates. (DOC) [file pone.0062427.s005.doc]

**Table S2. List of significant (p ≤ 0.00009) pairings of TH2 and TH3 Epitopes among Malawian Parasite Isolates.**

| **TH3-Type** | **TH2-Type** | **Observed** | **Predicted** | **SE** | **p_value** |
| --- | --- | --- | --- | --- | --- |
| 0 | 0 | 11 | 0.749 | 0.289 | <0.000001 |
| 0 | 8 | 4 | 0.545 | 0.233 | <0.000001 |
| 1 | 1 | 22 | 7.115 | 1.659 | <0.000001 |
| 2 | 3 | 26 | 4.094 | 1.013 | <0.000001 |
| 3 | 5 | 18 | 2.604 | 0.740 | <0.000001 |
| 4 | 9 | 13 | 1.162 | 0.403 | <0.000001 |
| 5 | 8 | 4 | 0.613 | 0.257 | <0.000001 |
| 5 | 18 | 4 | 0.306 | 0.168 | <0.000001 |
| 5 | 30 | 3 | 0.230 | 0.143 | <0.000001 |
| 5 | 23 | 1 | 0.077 | 0.079 | <0.000001 |
| 6 | 12 | 2 | 0.026 | 0.023 | <0.000001 |
| 6 | 29 | 1 | 0.013 | 0.015 | <0.000001 |
| 7 | 13 | 3 | 0.289 | 0.160 | <0.000001 |
| 7 | 15 | 3 | 0.217 | 0.135 | <0.000001 |
| 7 | 17 | 3 | 0.217 | 0.135 | <0.000001 |
| 7 | 16 | 2 | 0.145 | 0.108 | <0.000001 |
| 7 | 19 | 2 | 0.145 | 0.108 | <0.000001 |
| 7 | 21 | 1 | 0.072 | 0.074 | <0.000001 |
| 7 | 28 | 1 | 0.072 | 0.074 | <0.000001 |
| 8 | 14 | 8 | 0.421 | 0.187 | <0.000001 |
| 8 | 25 | 3 | 0.140 | 0.091 | <0.000001 |
| 9 | 27 | 3 | 0.064 | 0.046 | <0.000001 |
| 10 | 9 | 2 | 0.179 | 0.132 | <0.000001 |
| 11 | 27 | 2 | 0.043 | 0.035 | <0.000001 |
| 12 | 14 | 1 | 0.038 | 0.040 | <0.000001 |
| 13 | 24 | 1 | 0.026 | 0.028 | <0.000001 |
| 14 | 24 | 1 | 0.026 | 0.028 | <0.000001 |
| 1 | 2 | 17 | 5.498 | 1.431 | <0.000001 |
| 2 | 20 | 2 | 0.315 | 0.228 | <0.000001 |
| 3 | 7 | 10 | 3.472 | 0.898 | <0.000001 |
| 1 | 4 | 11 | 3.557 | 1.124 | <0.000001 |
| 1 | 6 | 10 | 3.234 | 1.067 | <0.000001 |
| 3 | 31 | 1 | 0.145 | 0.146 | <0.000001 |
| 3 | 32 | 1 | 0.145 | 0.146 | <0.000001 |
| 3 | 33 | 1 | 0.145 | 0.146 | <0.000001 |
| 5 | 7 | 5 | 1.838 | 0.561 | <0.000001 |
| 2 | 7 | 9 | 3.779 | 0.959 | <0.000001 |
| 1 | 3 | 0 | 8.409 | 1.830 | 0.000004 |
| 1 | 7 | 0 | 7.762 | 1.745 | 0.000009 |
| 3 | 3 | 0 | 3.762 | 0.949 | 0.000073 |
| 1 | 5 | 0 | 5.821 | 1.478 | 0.000082 |
