# Supplementary material for: Diversity of T Cell Epitopes in Plasmodium falciparum Circumsporozoite Protein Likely Due to Protein-Protein Interactions
Source: PLoS One. 2013 May 7;8(5):e62427. doi: 10.1371/journal.pone.0062427 (PMC3646838; doi:10.1371/journal.pone.0062427)
Supplement: Table S3 — List of significant (p≤0.003) haplotype pairings of TH2 and TH3 Epitopes among Gambian Parasite Isolates. (DOC) [file pone.0062427.s006.doc]

**Table S3: List of significant (p ≤ 0.003) haplotype pairings of TH2 and TH3 Epitopes among Gambian Parasite Isolates.**

| **TH3-Type** | **TH2-Type** | **Observed** | **Predicted** | **SE** | **p_value** |
| --- | --- | --- | --- | --- | --- |
| 2 | 3 | 11 | 2.357 | 0.932 | <0.000001 |
| 3 | 37 | 7 | 1.286 | 0.601 | <0.000001 |
| 7 | 36 | 3 | 0.321 | 0.223 | <0.000001 |
| 0 | 8 | 2 | 0.107 | 0.097 | <0.000001 |
| 4 | 9 | 1 | 0.054 | 0.061 | <0.000001 |
| 6 | 29 | 1 | 0.036 | 0.043 | <0.000001 |
| 5 | 23 | 1 | 0.018 | 0.025 | <0.000001 |
| 8 | 41 | 1 | 0.018 | 0.025 | <0.000001 |
| 14 | 40 | 1 | 0.018 | 0.025 | <0.000001 |
| 7 | 28 | 1 | 0.107 | 0.115 | <0.000001 |
| 7 | 31 | 1 | 0.107 | 0.115 | <0.000001 |
| 7 | 42 | 1 | 0.107 | 0.115 | <0.000001 |
| 0 | 9 | 1 | 0.161 | 0.129 | <0.000001 |
| 3 | 7 | 1 | 0.161 | 0.168 | 0.000001 |
| 3 | 39 | 1 | 0.161 | 0.168 | 0.000001 |
| 1 | 6 | 6 | 2.250 | 0.997 | 0.000170 |
| 1 | 1 | 5 | 1.875 | 0.899 | 0.000507 |
| 1 | 2 | 4 | 1.500 | 0.793 | 0.001627 |
