# Supplementary material for: Diversity of T Cell Epitopes in Plasmodium falciparum Circumsporozoite Protein Likely Due to Protein-Protein Interactions
Source: PLoS One. 2013 May 7;8(5):e62427. doi: 10.1371/journal.pone.0062427 (PMC3646838; doi:10.1371/journal.pone.0062427)
Supplement: Table S4 — Average and Standard Deviation of Free Energy of Mutation. (DOC) [file pone.0062427.s007.doc]

**Table S4. Average and Standard Deviation of Free Energy of Mutation.** The underlying data for each amino acid type averaged across the observed positions within the reference structure (the average energy in kcal/mol as obtained from MUMBO calculations). The average energy for all amino acids and one standard deviation for each type of amino acid to mutate to the 19 possibilities. N is the number of observed occurrences within the reference crystal structure included.

| ASP,N=4 | avg | stdev |
| --- | --- | --- |
| ASN | -0.64 | 0.98 |
| GLU | 2.08 | 2.88 |
| GLN | 0.68 | 2.78 |
| SER | -0.59 | 2.26 |
| THR | -4.15 | 2.41 |
| CYS | -1.76 | 1.91 |
| MET | -0.83 | 3.02 |
| GLY | -0.68 | 1.73 |
| ALA | -4.21 | 1.98 |
| VAL | -6.54 | 2.66 |
| LEU | -3.76 | 6.63 |
| ILE | -5.31 | 5.21 |
| HIS | -1.03 | 4.61 |
| LYS | 1.25 | 3.58 |
| ARG | 6.95 | 3.47 |
| PHE | -3.55 | 3.24 |
| TYR | -7.16 | 5.07 |
| TRP | -3.07 | 8.91 |
| PRO | 14.77 | 24.15 |
|  |  |  |
| ASN,N=5 | avg | stdev |
| ASP | 2.6 | 1.49 |
| GLU | 2.33 | 1.71 |
| GLN | -0.07 | 1.24 |
| SER | 0.08 | 1.13 |
| THR | -2.55 | 2.27 |
| CYS | -1.36 | 1.01 |
| MET | -0.43 | 1.52 |
| GLY | -0.56 | 1.08 |
| ALA | -3.01 | 1.04 |
| VAL | -5.48 | 3.9 |
| LEU | -5.74 | 2.07 |
| ILE | -6.08 | 3.99 |
| HIS | -0.67 | 2 |
| LYS | 0.36 | 1.03 |
| ARG | 6.05 | 1.14 |
| PHE | -7.19 | 2.43 |
| TYR | -6.96 | 2 |
| TRP | -6.8 | 3.21 |
| PRO | 2.07 | 13.95 |
|  |  |  |
| GLU,N=7 | avg | stdev |
| ASP | 1.78 | 2.84 |
| ASN | -1.41 | 2.77 |
| GLN | -3.03 | 2.3 |
| SER | -1.49 | 2.36 |
| THR | -4.49 | 2.45 |
| CYS | -3.18 | 2.36 |
| MET | -2.78 | 2.26 |
| GLY | -2.09 | 2.95 |
| ALA | -5.45 | 2.27 |
| VAL | -8.35 | 2.47 |
| LEU | -9.18 | 2.15 |
| ILE | -8.17 | 4.17 |
| HIS | -3.12 | 5.15 |
| LYS | -1.92 | 4.88 |
| ARG | 4.15 | 5.08 |
| PHE | -8.05 | 4.64 |
| TYR | -7.41 | 6.43 |
| TRP | -8.38 | 1.78 |
| PRO | 10.26 | 19.86 |
|  |  |  |
| GLN,N=2 | avg | stdev |
| ASP | 9.66 | 6.64 |
| ASN | 5.09 | 2.93 |
| GLU | 3.85 | 1.81 |
| SER | 0.18 | 3.98 |
| THR | -0.89 | 0.05 |
| CYS | 0.65 | 1.66 |
| MET | -1.89 | 0.73 |
| GLY | 0.62 | 3.63 |
| ALA | -4.85 | 1.84 |
| VAL | -7.73 | 1.84 |
| LEU | -2.7 | 6.26 |
| ILE | -10.75 | 3.34 |
| VAL | -7.73 | 1.84 |
| HIS | 21.35 | 31.05 |
| LYS | 0.68 | 0.09 |
| ARG | 7.48 | 0.41 |
| PHE | 46.46 | 69.66 |
| TYR | 52.11 | 78.88 |
| TRP | 32.53 | 54.04 |
| PRO | 22.34 | 13.23 |
|  |  |  |
| SER,N=6 | avg | stdev |
| ASP | 10.74 | 7.53 |
| ASN | 4.72 | 5.4 |
| GLU | 8.45 | 7.89 |
| GLN | 1.31 | 5.28 |
| THR | -1.84 | 2.32 |
| CYS | 0.28 | 2.64 |
| MET | 0.8 | 4.88 |
| GLY | 1 | 1.72 |
| ALA | -3.78 | 1.61 |
| VAL | -5.01 | 4.41 |
| LEU | 3.79 | 12.33 |
| ILE | -2.87 | 7.66 |
| HIS | 10.44 | 18.89 |
| LYS | 5.23 | 7.02 |
| ARG | 11.48 | 9.46 |
| PHE | 8.22 | 21.59 |
| TYR | 18.53 | 47.17 |
| TRP | 11.51 | 26.74 |
| PRO | 23.14 | 28.17 |
|  |  |  |
| THR,N=2 | avg | stdev |
| ASP | 8.41 | 2.71 |
| ASN | 5.83 | 2.43 |
| GLU | 8.82 | 5.07 |
| GLN | 4.25 | 1.42 |
| SER | 3.81 | 2.05 |
| CYS | 3.16 | 2.24 |
| MET | 2.84 | 1.59 |
| GLY | 4.3 | 3.01 |
| ALA | 1.54 | 1.68 |
| VAL | -3.43 | 0.74 |
| LEU | -2.63 | 0.25 |
| ILE | -4.12 | 0.81 |
| HIS | 2.87 | 1.58 |
| LYS | 5.85 | 3.7 |
| ARG | 9.7 | 0.32 |
| PHE | -1.4 | 3.55 |
| TYR | -1.02 | 4.18 |
| TRP | -4.01 | 20.9 |
| PRO | -1.42 | 2.25 |
|  |  |  |
| CYS,N=4 | avg | stdev |
| ASP | 8.42 | 2.13 |
| ASN | 4.63 | 1.69 |
| GLU | 7.34 | 4.34 |
| GLN | 3.43 | 2.84 |
| SER | 0.73 | 0.78 |
| THR | -2.34 | 2.84 |
| MET | 3.25 | 4.08 |
| GLY | 0.85 | 1.62 |
| ALA | -3.97 | 1.72 |
| VAL | -5.77 | 2.28 |
| LEU | 4.71 | 11.52 |
| ILE | -2.92 | 5.15 |
| HIS | 6.65 | 10.76 |
| LYS | 4.22 | 3.84 |
| ARG | 8.65 | 3.07 |
| PHE | 3.1 | 13.04 |
| TYR | 2.75 | 13.15 |
| TRP | 7.22 | 11.2 |
| PRO | -2.36 | 7.5 |
|  |  |  |
| MET,N=1 | avg | stdev |
| ASP | 16.79 | 0 |
| ASN | 9.99 | 0 |
| GLU | 8.37 | 0 |
| GLN | 2.36 | 0 |
| SER | 8.58 | 0 |
| THR | 6.52 | 0 |
| CYS | 4.86 | 0 |
| GLY | 8.03 | 0 |
| ALA | 1.74 | 0 |
| VAL | 17.88 | 0 |
| LEU | 0.48 | 0 |
| ILE | 11.69 | 0 |
| HIS | 12.1 | 0 |
| LYS | 4.62 | 0 |
| ARG | 10.83 | 0 |
| PHE | 27.14 | 0 |
| TYR | 83.83 | 0 |
| TRP | 31.21 | 0 |
| PRO | 22.26 | 0 |
|  |  |  |
| GLY,N=3 | avg | stdev |
| ASP | 16.66 | 21.66 |
| ASN | 13.06 | 19.87 |
| GLU | 14.87 | 22.91 |
| GLN | 13.36 | 21.92 |
| SER | 6.63 | 9.89 |
| THR | 9.96 | 11.47 |
| CYS | 4.43 | 8.64 |
| MET | 16.09 | 28.9 |
| ALA | 1.15 | 5.68 |
| VAL | 23.71 | 29.04 |
| LEU | 8.23 | 25.59 |
| ILE | 18.41 | 25.63 |
| HIS | 45.86 | 59.36 |
| LYS | 24.84 | 39.61 |
| ARG | 30.7 | 40.16 |
| PHE | 54.76 | 79.69 |
| TYR | 65.21 | 103.04 |
| TRP | 64.68 | 107 |
| PRO | 13.55 | 15.92 |
|  |  |  |
| ALA,N=2 | avg | stdev |
| ASP | 8.43 | 3.17 |
| ASN | 5.81 | 4.51 |
| GLU | 8.2 | 2.38 |
| GLN | 4.9 | 1.44 |
| SER | 3.07 | 1.01 |
| THR | 4.29 | 4.12 |
| CYS | 3.3 | 1.93 |
| MET | 5.83 | 2.73 |
| GLY | 2.87 | 1.45 |
| VAL | -1.6 | 0.04 |
| LEU | 1.31 | 3.1 |
| ILE | -1.69 | 0.39 |
| HIS | 4.04 | 0.99 |
| LYS | 3.85 | 0.36 |
| ARG | 9.94 | 0.15 |
| PHE | -1.04 | 1.36 |
| TYR | -1.62 | 0.75 |
| TRP | 1.42 | 2.46 |
| PRO | 28.1 | 39.69 |
|  |  |  |
| VAL,N=3 | avg | stdev |
| ASP | 18.48 | 8.57 |
| ASN | 12.57 | 3.43 |
| GLU | 17.67 | 9.13 |
| GLN | 12.65 | 6.76 |
| SER | 13.45 | 4.55 |
| THR | 7.5 | 3.88 |
| CYS | 11.03 | 3.69 |
| MET | 8.46 | 1.78 |
| GLY | 14 | 5.32 |
| ALA | 8.79 | 3.68 |
| LEU | 4.38 | 5.22 |
| ILE | 4.26 | 6.24 |
| HIS | 16.09 | 16.86 |
| LYS | 16.66 | 10.66 |
| ARG | 23.57 | 9.18 |
| PHE | 13.82 | 18.46 |
| TYR | 19.69 | 28.48 |
| TRP | 13.4 | 16.97 |
| PRO | 6.18 | 2.49 |
|  |  |  |
| LEU,N=3 | avg | stdev |
| ASP | 25.95 | 1.26 |
| ASN | 16.12 | 2.01 |
| GLU | 22.6 | 0.57 |
| GLN | 12.65 | 0.83 |
| SER | 15.93 | 2.07 |
| THR | 9.67 | 2.15 |
| CYS | 13.96 | 1.78 |
| MET | 10.61 | 3.33 |
| GLY | 16.27 | 2.36 |
| ALA | 11.49 | 1.56 |
| VAL | 2.81 | 1.57 |
| ILE | -0.1 | 1.9 |
| HIS | 13.59 | 4.75 |
| LYS | 18.01 | 0.93 |
| ARG | 24.15 | 2.29 |
| PHE | 3.62 | 6.94 |
| TYR | 8.5 | 8.05 |
| TRP | 10.53 | 7.52 |
| PRO | 50.02 | 26.59 |
|  |  |  |
| ILE,N=6 | avg | stdev |
| ASP | 21.87 | 5.75 |
| ASN | 14.58 | 3.94 |
| GLU | 19.1 | 8.08 |
| GLN | 12.31 | 5.7 |
| SER | 14.25 | 4.18 |
| THR | 8.48 | 3.83 |
| CYS | 12.49 | 4.43 |
| MET | 10.11 | 4.78 |
| GLY | 14.48 | 4.65 |
| ALA | 9.24 | 3.75 |
| VAL | 0.9 | 3.93 |
| LEU | 5.52 | 4.55 |
| HIS | 21.8 | 16.24 |
| LYS | 17 | 7.09 |
| ARG | 22.31 | 6.37 |
| PHE | 20.41 | 31.7 |
| TYR | 48.83 | 76.67 |
| TRP | 18.57 | 31.57 |
| PRO | 26.82 | 19.03 |
|  |  |  |
| HIS,N=2 | avg | stdev |
| ASP | 1.67 | 4.43 |
| ASN | -0.21 | 1.39 |
| GLU | 1.13 | 4.58 |
| GLN | -0.87 | 1.92 |
| SER | -0.76 | 1.95 |
| THR | -2.1 | 1.6 |
| CYS | -1.25 | 1.86 |
| MET | -0.49 | 0.21 |
| GLY | -1.19 | 3.77 |
| ALA | -3.44 | 1.61 |
| VAL | -4.82 | 0.41 |
| LEU | -5.46 | 0.51 |
| ILE | -5.08 | 0.27 |
| LYS | 0.92 | 0.34 |
| ARG | 7.08 | 0.35 |
| PHE | -6.07 | 0.36 |
| TYR | -6.05 | 0.83 |
| TRP | -6.41 | 3.12 |
| PRO | -0.14 | 5.61 |
|  |  |  |
| LYS,N=7 | avg | stdev |
| ASP | 8.28 | 5.85 |
| ASN | 1.79 | 3.5 |
| GLU | 6.98 | 4.04 |
| GLN | 1.43 | 2.32 |
| SER | 2.12 | 3.59 |
| THR | -1.95 | 4.35 |
| CYS | 0.37 | 3.4 |
| MET | -0.62 | 3.16 |
| GLY | 2.03 | 3.9 |
| ALA | -2.14 | 3.09 |
| VAL | -6.11 | 3.76 |
| LEU | -4.86 | 1.4 |
| ILE | -7.19 | 4.23 |
| HIS | -1.19 | 2.35 |
| ARG | 6.8 | 1.79 |
| PHE | -7.45 | 2.21 |
| TYR | -7.33 | 2.21 |
| TRP | -7.32 | 4.45 |
| PRO | 19.88 | 26.45 |
|  |  |  |
| ARG,N=1 | avg | stdev |
| ASP | 14.25 | 0 |
| ASN | 2.75 | 0 |
| GLU | 3.99 | 0 |
| GLN | -5.77 | 0 |
| SER | 1.29 | 0 |
| THR | -3.12 | 0 |
| CYS | -0.13 | 0 |
| MET | -0.13 | 0 |
| GLY | 5.46 | 0 |
| ALA | -3.38 | 0 |
| VAL | 8.18 | 0 |
| LEU | -3.58 | 0 |
| ILE | 7.43 | 0 |
| HIS | 14.47 | 0 |
| LYS | -2.56 | 0 |
| PHE | 7.13 | 0 |
| TYR | 8.9 | 0 |
| TRP | 8.69 | 0 |
| PRO | 4.33 | 0 |
|  |  |  |
| PHE,N=1 |  |  |
| ASP | 17.34 | 0 |
| ASN | 14.75 | 0 |
| GLU | 14.17 | 0 |
| GLN | 12.78 | 0 |
| SER | 11.67 | 0 |
| THR | 12.47 | 0 |
| CYS | 11.74 | 0 |
| MET | 11.26 | 0 |
| GLY | 12.56 | 0 |
| ALA | 10.63 | 0 |
| VAL | 9.78 | 0 |
| LEU | 5.95 | 0 |
| ILE | 9.14 | 0 |
| HIS | 9.07 | 0 |
| LYS | 11.65 | 0 |
| ARG | 18.31 | 0 |
| TYR | 0.29 | 0 |
| TRP | 2.5 | 0 |
| PRO | 30.74 | 0 |
|  |  |  |
| TRP,N=1 | avg | stdev |
| ASP | 25.62 | 0 |
| ASN | 21.53 | 0 |
| GLU | 24.08 | 0 |
| GLN | 21.19 | 0 |
| SER | 20.34 | 0 |
| THR | 16.85 | 0 |
| CYS | 19.02 | 0 |
| MET | 16.55 | 0 |
| GLY | 18.89 | 0 |
| ALA | 15.72 | 0 |
| VAL | 13.33 | 0 |
| LEU | 10.85 | 0 |
| ILE | 13.74 | 0 |
| HIS | 20.93 | 0 |
| LYS | 24.76 | 0 |
| ARG | 29.47 | 0 |
| PHE | 9.4 | 0 |
| TYR | 9.35 | 0 |
| PRO | 13.55 | 0 |
|  |  |  |
| TYR,N=3 | avg | stdev |
| ASP | 18.02 | 9.3 |
| ASN | 14.5 | 7.01 |
| GLU | 17.55 | 8.56 |
| GLN | 13.14 | 6.11 |
| SER | 13.98 | 4.86 |
| THR | 9.77 | 3.57 |
| CYS | 12.52 | 4.7 |
| MET | 14.16 | 5.28 |
| GLY | 13.17 | 5.86 |
| ALA | 10.01 | 4.76 |
| VAL | 5.6 | 4.39 |
| LEU | 7.79 | 4.91 |
| ILE | 6.97 | 5.19 |
| HIS | 10.66 | 3.87 |
| LYS | 18.12 | 8.63 |
| ARG | 23.12 | 6.68 |
| PHE | -0.16 | 0.39 |
| TRP | 2.04 | 9.82 |
| PRO | 21.69 | 22.06 |
|  |  |  |
| PRO,N=5 | avg | stdev |
| ASP | 9.53 | 1.55 |
| ASN | 5.92 | 1.6 |
| GLU | 9.49 | 1.43 |
| GLN | 4.78 | 1.65 |
| SER | 5.58 | 1.79 |
| THR | 2.69 | 0.43 |
| CYS | 4.24 | 1.51 |
| MET | 4.55 | 2.33 |
| GLY | 4.82 | 1.47 |
| ALA | 2.12 | 1.46 |
| VAL | 0.04 | 2.09 |
| LEU | -1.38 | 2.79 |
| ILE | -0.29 | 3.11 |
| HIS | 4.75 | 2.87 |
| LYS | 6.27 | 5.12 |
| ARG | 11.87 | 4.74 |
| PHE | 1.86 | 7.86 |
| TYR | 3.03 | 9.97 |
| TRP | 3.06 | 8.19 |
